# MicroRNA-29a in Osteoblasts Represses High-Fat Diet-Mediated Osteoporosis and Body Adiposis through Targeting Leptin

**DOI:** 10.3390/ijms22179135

**Published:** 2021-08-24

**Authors:** Wei-Shiung Lian, Re-Wen Wu, Yu-Shan Chen, Jih-Yang Ko, Shao-Yu Wang, Holger Jahr, Feng-Sheng Wang

**Affiliations:** 1 Core Laboratory for Phenomics and Diagnostic, Department of Medical Research, Chang Gung University College of Medicine, Kaohsiung Chang Gung Memorial Hospital, Kaohsiung 83301, Taiwan; lianws@gmail.com (W.-S.L.); ggyy58720240@gmail.com (Y.-S.C.); vip690221@gmail.com (S.-Y.W.); 2 Center for Mitochondrial Research and Medicine, Kaohsiung Chang Gung Memorial Hospital, Kaohsiung 83301, Taiwan; 3 Department of Orthopedic Surgery, Chang Gung University College of Medicine, Kaohsiung Chang Gung Memorial Hospital, Kaohsiung 83301, Taiwan; ray4595@cgmh.org.tw (R.-W.W.); kojy@cgmh.org.tw (J.-Y.K.); 4 Department of Anatomy and Cell Biology, University Hospital RWTH Aachen, 52074 Aachen, Germany; h.jahr@masstrichtuniversity.nl; 5 Department of Orthopedic Surgery, Maastricht University Medical Center, 6229ER Maastricht, The Netherlands

**Keywords:** microRNA-29a, bone loss, visceral fat deposit, heat production, leptin

## Abstract

Skeletal tissue involves systemic adipose tissue metabolism and energy expenditure. MicroRNA signaling controls high-fat diet (HFD)-induced bone and fat homeostasis dysregulation remains uncertain. This study revealed that transgenic overexpression of miR-29a under control of osteocalcin promoter in osteoblasts (miR-29aTg) attenuated HFD-mediated body overweight, hyperglycemia, and hypercholesterolemia. HFD-fed miR-29aTg mice showed less bone mass loss, fatty marrow, and visceral fat mass together with increased subscapular brown fat mass than HFD-fed wild-type mice. HFD-induced O_2_ underconsumption, respiratory quotient repression, and heat underproduction were attenuated in miR-29aTg mice. In vitro, miR-29a overexpression repressed transcriptomic landscapes of the adipocytokine signaling pathway, fatty acid metabolism, and lipid transport, etc., of bone marrow mesenchymal progenitor cells. Forced miR-29a expression promoted osteogenic differentiation but inhibited adipocyte formation. miR-29a signaling promoted brown/beige adipocyte markers Ucp-1, Pgc-1α, P2rx5, and Pat2 expression and inhibited white adipocyte markers Tcf21 and Hoxc9 expression. The microRNA also reduced peroxisome formation and leptin expression during adipocyte formation and downregulated HFD-induced leptin expression in bone tissue. Taken together, miR-29a controlled leptin signaling and brown/beige adipocyte formation of osteogenic progenitor cells to preserve bone anabolism, which reversed HFD-induced energy underutilization and visceral fat overproduction. This study sheds light on a new molecular mechanism by which bone integrity counteracts HFD-induced whole-body fat overproduction.

## 1. Introduction

Bone tissue plays an endocrine role in producing a plethora of molecules, including hormones, growth factors, neuropeptides, and exosomes, regulating marrow fat homeostasis, peripheral adipose metabolism, and energy expenditure [1,2]. Osteoporosis correlates with increased fat mass in the elderly [3] and the development of abdomen adiposity [4]. Expanding evidence reveals low bone mass together with high blood glucose levels and energy metabolism in mice lacking the Wnt signaling component β-catenin in osteoblasts [5]. Mice deficient in Dickkopf-1, an inhibitor for the Wnt pathway, in osteogenic progenitor cells exhibit body overweight and increased white fat pad [6]. Mesenchymal progenitor cell-specific PPARγ knockout mice develop phenotypes with high cortical bone microstructure and low epididymal fat mass [7].

High-fat diet (HFD) induces glucose, energy, and fat metabolism dysregulation, accelerating tissue deterioration and dysfunction [8]. Accumulating studies have revealed osteogenesis downregulation [9], fatty marrow, bone mass loss [10], and biomechanical property repression [11] in mice upon HFD feeding. Oxidative stress [12], inflammatory cytokine overproduction [13], and Wnt signaling loss [14] aggravate the development of HFD-induced bone loss. In addition, epigenetic pathways, including histone lysine demethylase [15], RNA demethylase [16], and long non-coding RNA, in osteogenic cells [17] regulates HFD-induced bone mass loss and peripheral fat metabolism.

Of epigenetic pathways, small non-coding microRNAs (miR) bind to 3′-untranslated regions of mRNA targets, disrupting protein translation to control physiological or pathological activities [18]. A plethora of microRNAs in the bone microenvironment involve bone metabolism and marrow fat formation during osteoporotic skeleton development [19]. For example, miR-146a knockout mice show fewer age-induced osteoporosis signs together with activated Wnt signaling and increased osteoblast differentiation [20]. miR-378a-3p mediates low magnitude vibration-induced increases in bone formation, delaying osteoporosis development [21]. Mice overexpressing miR-188 display severe osteoporosis and marrow adiposis [22]. Increasing evidence reveals that the miR-29a family modulates osteogenic or osteoclastogenic progenitor cell function. miR-29a represses oxidative stress, compromising the senescence program of mesenchymal stem cells [23]. Mice overexpressing miR-29-3p decoy inhibitor have high osteoclastic resorption in trabecular bone [24]. miR-29-3p inhibitor interrupts osteoblastic differentiation but compromises parathyroid hormone-mediated bone formation [25]. miR-29a transgenic mice develop high bone mass and trabecular network [26]. However, the biological function of miR-29a signaling to HFD-mediated bone and peripheral adipose metabolism remains uncertain.

This study aimed to use osteoblast-specific miR-29a transgenic mice to examine whether bone mass or visceral fat mass or in vivo metabolism were affected upon HFD feeding and characterized whether the microRNA changed white or brown adipogenic differentiation capacity or peroxisome formation.

## 2. Results

### 2.1. miR-29a Reversed HFD-Induced Bone Mass Loss and Marrow Adiposity

**Figure 1:**
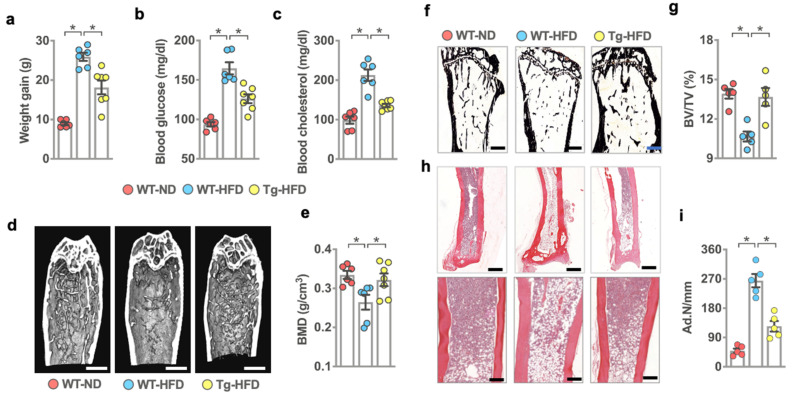
Analysis of serum biochemistry, bone mass, and histology of wild-type and miR-29aTg mice upon standard rodent chow (ND) and high-fat diet (HFD) feeding. HFD-mediated increases in body weight gain (**a**), serum cholesterol (**b**), and blood glucose levels (**c**) were compromised in miR-29aTg mice. Sagittal views of μCT images showing scarce trabecular bone network in HFD-fed WT mice and dense bone microstructure in HFD-fed miR-29Tg mice (**d**); scale bar, 5 mm. miR-29a overexpression attenuated HFD-induced loss in bone mineral density (**e**). Investigations (mean ± S.E.) are calculated from 6–7 mice. miR-29a overexpression compromised HFD-mediated sparse trabecular morphology (**f**), BV/TV loss (**g**) (scale bar, 40 µm), marrow adiposity (**h**) (scale bars, 100 µm in higher panels and 40 µm in lower panels), and Ad.N (**i**). Mean ± S.E. is calculated from 5 mice. * *p* < 0.05 upon ANOVA test and Bonferroni post hoc test. ND, standard rodent chow; HFD, high-fat diet.

We characterized what role miR-29a signaling in bone-forming cells may play in bone mass and visceral fat formation of HFD-fed mice. To this end, wild-type mice (WT) and osteoblast-specific miR-29a transgenic mice (miR-29Tg; under control of osteocalcin promoter), which had a high bone mass phenotype [26], were fed with standard rodent chow (ND, 10 kcal% fat) or high-fat diet (HFD; 60 kcal% fat) for six consecutive months. HFD-fed WT mice had significant increases in body weight (Figure 1a), serum cholesterol (Figure 1b), and blood glucose levels around 149–188 mg/dl (Figure 1c) compared to ND-fed WT mice. HFD-induced body overweight and increases in serum cholesterol and blood glucose were compromised in miR-29aTg mice.

Sagittal views of µCT images revealed scanty and disarranged trabecular bone microstructure (Figure 1d) together with significant decreases in bone mineral density (Figure 1e) in WT mice upon HFD feeding for 6 months. HFD-fed miR-29aTg mice showed well-entwined trabecular bone architecture and moderate loss in bone mass. Consistent with µCT analysis, the osteoporotic skeleton in HFD-fed WT mice showed poor trabecular morphology (Figure 1f) and significant decreases in trabecular bone volume (BV/TV; Figure 1g), whereas plenty of marrow adipocytes (Figure 1h), as evident from increased adipocyte number (Ad.N; Figure 1i), were present in these specimens. Bone tissue in HFD-fed miR-29aTg mice developed mild loss in trabecular bone volume and less marrow fat formation as compared to HFD-fed WT mice.

### 2.2. miR-29a Improved Visceral Fat and Subscapular Brown Fat Mass

**Figure 2:**
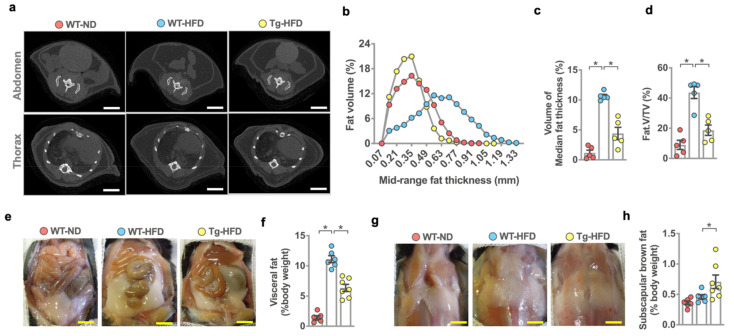
Analysis of adipose tissue of wild-type and miR-29aTg mice upon standard rodent chow (ND) and high-fat diet (HFD) feeding. μCT images of the abdomen fat and subcutaneous fat in the thorax compartment (**a**); scale bar, 15 mm. miR-29a reversed HFD-induced overall fat thickness (**b**), median fat thickness (**c**) and Fat.V/TV (**d**). Investigations (mean ± S.E.) are calculated from 5 mice. Images of visceral adipose tissue (**e**); scale bar, 18 mm. miR-29a attenuated HFD-induced visceral fat weight (**f**). Images of subscapular brown fat tissue (**g**), scale bar; 18 mm. HFD-fed miR-29a mice showed high subscapular brown fat mass (**h**). Mean ± S.E. is calculated from 6–7 mice. * *p* < 0.05 upon ANOVA test and Bonferroni post hoc test. ND, standard rodent chow; HFD, high-fat diet.

The investigations of moderate body weight gain in HFD-fed miR-29aTg mice prompted us to ask whether systemic adipose formation was changed in these animals upon HFD feeding. We adopted microcomputed tomography (μCT) [27,28] to characterize and quantify whole-body adipose tissue distribution. Cross-sectional µCT radiographs revealed that HFD-fed WT mice developed capacious adipose tissue in the abdomen, thorax, and subcutaneous compartments compared to ND-fed WT mice (Figure 2a). HFD-fed miR-29aTg mice showed moderate adiposity in these compartments. Likewise, adipose tissue thickness, including overall adipose thickness (Figure 2b) and median adipose thickness (Figure 2c) together with adipose volume (Fat.V/TV) (Figure 2d), were significantly upregulated in HFD-fed WT mice. miR-29Tg mice had less adipose thickness and fat volume than WT mice upon HFD feeding.

We investigated whether white fat or brown adipose development was affected in the development of HFD-induced adiposis. HFD-fed WT mice showed voluminous visceral fat formation (Figure 2e) together with significant increases in visceral adipose weight (Figure 2f), whereas subscapular brown adipose development (Figure 2g) and of such tissue weight (Figure 2h) were unaffected as compared to ND-fed WT mice. HFD-fed miR-29aTg mice had less visceral adipose formation than HFD-fed WT mice. Interestingly, subscapular brown fat mass was significantly increased in miR-29aTg mice upon HFD feeding.

### 2.3. miR-29a Affected In Vivo Metabolism and Heat Production

**Figure 3:**
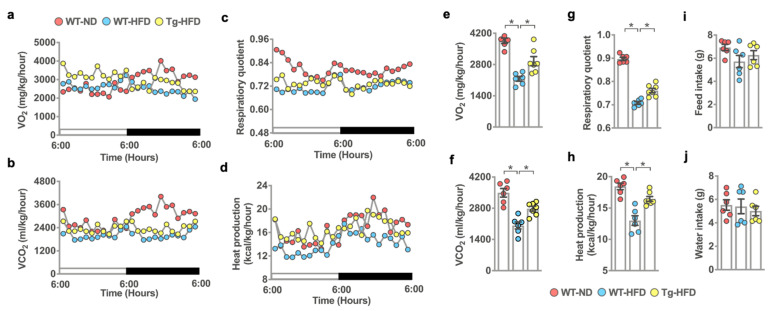
Effects of miR-29a overexpression on in vivo metabolism of HFD-fed mice. Kinetic changes of O_2_ consumption (**a**), CO_2_ production (**b**), respiration quotient (**c**), and heat production (**d**) of animals accommodated individually in Phenomaster metabolic cages at 23 °C for 24 h. miR-29a overexpression reversed HFD-induced O_2_ underconsumption (**e**), CO_2_ underproduction (**f**), respiratory quotient repression (**g**), and heat underproduction (**h**), whereas feed (**i**) and water intake (**j**) were unaffected. Mean ± S.E. is calculated from 6 mice. * *p* < 0.05 upon ANOVA test and Bonferroni post hoc test. ND, standard rodent chow; HFD, high-fat diet.

We wondered whether the changes in body fat deposition correlated with animals’ energy metabolism. To this end, we utilized the Automated Home Cage Phenotyping System to quantify in vivo metabolic profiles, including O_2_ consumption (Figure 3a), CO_2_ exhalation (Figure 3b), respiratory quotient (Figure 3c), and heat production (Figure 3d) at 23 °C from 6:00 am to 6:00 pm. HFD-fed WT mice had less O_2_ consumption (Figure 3e), CO_2_ production (Figure 3f), respiratory quotient (Figure 3g), and heat production (Figure 3h) compared to ND-fed WT mice. Of note, the extent of HFD-induced metabolism downregulation was attenuated in miR-29aTg mice. Feed (Figure 3i) and water intake (Figure 3j) were unaffected in three groups. These investigations suggested that HFD-fed miR-29a mice had more fat utilization or energy consumption than HFD-fed WT mice.

### 2.4. miR-29a Regulated Transcriptomic Landscapes of Fat Metabolism

**Figure 4:**
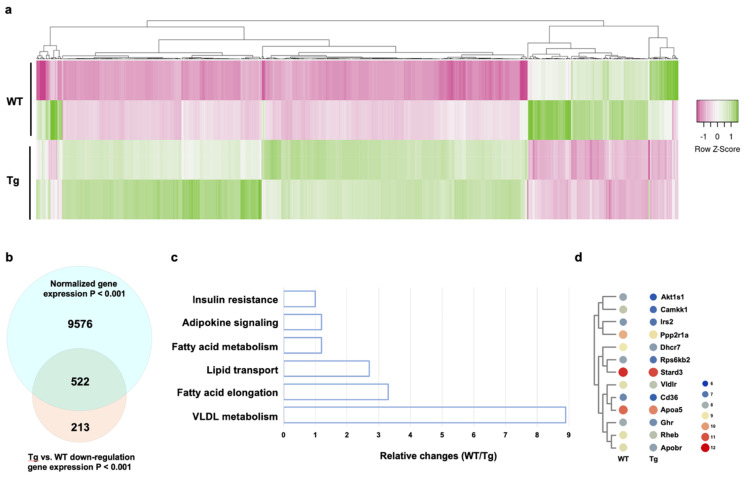
Whole-genome microarray analysis of transcriptomic profiles of miR-29aTg and wild-type bone marrow mesenchymal cells. Heatmap showing differential gene transcription of miR-29aTg and WT cells (**a**). Venn diagram showing 213 gene transcription repression in miR-29aTg cells (**b**). KEGG pathway analysis revealed that miR-29a downregulated gene transcription, which involved fat, fatty acid, lipid transport, and lipoprotein metabolism (**c**). Heatmap of transcriptome involving fatty acid and lipid metabolism (**d**). Microarray analysis was performed in 3 experiments.

Given that gain of miR-29a signaling in bone tissue reversed HFD-mediated whole-body fat production and energy metabolism dysregulation, we examined whether fat metabolism was changed in the bone microenvironment. Whole-genome microarray was utilized to characterize transcriptomic profiles (Figure 4a) of bone marrow mesenchymal cells isolated from WT and miR-29Tg mice. The 9789 gene transcription was significantly changed (*p* < 0.001) in the miR-29aTg group. Transgenic overexpression of miR-29a induced 213 gene transcription repression (Figure 4b). These genes involved insulin resistance, adipocytokine signaling, fatty acid metabolism, lipid transport, fatty acid elongation, and very-low-density lipoprotein (VLDL) metabolism (Figure 4c), as evidenced in KEGG pathway bioinformatics computation. In addition, miR-29a signaling inhibited a plethora of gene transcription for fatty acid/lipid metabolism, including Akt1 substrate 1 (Akt1s1), insulin receptor substrate 2 (Irs2), protein phosphatase 2, regulatory subunit 1a (Ppp2r1a), very-low-density lipoprotein receptor (Vldlr), CD36, apolipoprotein a5 (Apoa5), growth hormone receptor (Ghr), and apolipoprotein b receptor (Apobr; Figure 4d).

### 2.5. miR-29a Controlled Osteogenesis and Brown Adipogenesis

**Figure 5:**
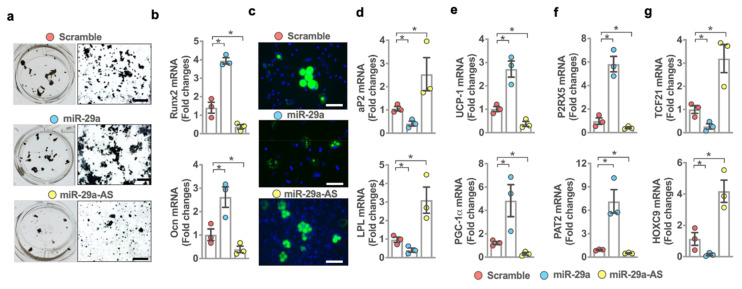
Effects of miR-29a mimics and antisense oligonucleotides on osteogenic differentiation and adipocyte formation of bone marrow mesenchymal stem cells. Forced miR-29a expression promoted mineralized matrix formation (**a**) (von Kossa staining; scale bar, 40 µm) together with increased Runx2 and Ocn expression (**b**). miR-29a mimetics repressed fluorescence adipocyte formation (**c**) (fluorescence Nile red staining; scale bar, 8 µm) and reduced aP2 and LPL expression (**d**). miR-29a mimetics promoted Ucp-1, Pgc-1α (**e**), P2rx5, and Pat2 (**f**) expression in mesenchymal stem cells incubated in brown adipogenic medium, whereas Tcf21 and Hoxc9 (**g**) expression were repressed in cell cultures grown in white adipogenic medium. miR-29a antisense oligonucleotide (miR-29a-AS) inhibited osteogenesis and brown adipocyte marker expression but upregulated adipocyte formation together with upregulated white adipocyte marker expression. Mean ± S.E. is calculated from 3 experiments. * *p* < 0.05 upon ANOVA test and Bonferroni post hoc test. Scramble, scrambled control; miR-29a, miR-29a mimics; miR-29a-AS, miR-29a antisense oligonucleotides.

Bone marrow adipose composition correlated with the development of osteoporosis and obesity [29,30]. We incubated bone marrow mesenchymal stem cells in osteogenic or adipogenic differentiation media and investigated whether miR-29a signaling changed osteogenesis or adipogenesis. Forced miR-29a expression by miR-29a mimetics promoted mineralized extracellular matrix accumulation as evidenced in von Kossa cytochemical staining (Figure 5a) together with significant increases in osteogenic markers Runx2 and osteocalcin expression (Figure 5b), as compared to the scrambled control group. Gain of miR-29a function reduced adipocyte formation (Figure 5c) as evident from fluorescence Nile red staining and downregulated adipocyte marker expression below baseline, including fatty acid-binding protein 4 (aP2) and lipoprotein lipase (Lpl) (Figure 5d). miR-29a knockdown by antisense oligonucleotides inhibited osteogenic differentiation capacity but upregulated adipocytic activity of bone marrow mesenchymal stem cells.

The analysis of adipogenic differentiation of mesenchymal stem cells prompted us to examine whether miR-29a signaling changed white or brown adipocyte formation. Mesenchymal stem cells were incubated in white and brown adipogenic conditions, respectively. Interestingly, the gain of miR-29a function promoted brown adipocyte marker expression, including uncoupling protein 1 (Ucp-1), peroxisome proliferative activated receptor-gamma, coactivator 1 alpha (Pgc-1α; Figure 5e), purinergic receptor P2X5 (P2rx5), and proton assistant amino acid transporter 2 (Pat2; Figure 5f), in mesenchymal stem cells grown in a brown adipogenic condition. miR-29a mimetics repressed white adipocyte marker expression, such as transcription factor 21 (Tcf21) and homeobox c9 (Hoxc9; Figure 5g), in cell cultures incubated in white adipogenic media. Loss of miR-29a function reduced brown adipocyte marker expression but upregulated white adipocyte marker expression.

### 2.6. miR-29a Accelerated Peroxisome Loss during Adipocyte Formation

**Figure 6:**
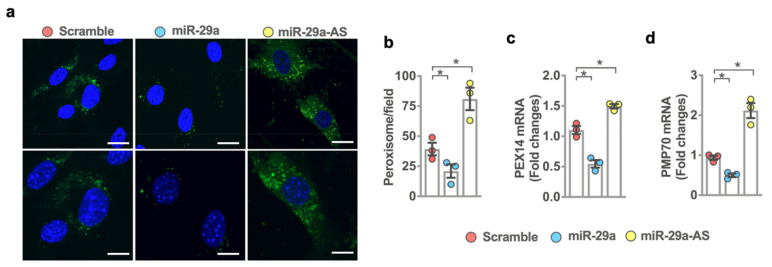
Analysis of peroxisome biogenesis in miR-29a mimic or antisense oligonucleotide-treated bone marrow mesenchymal stem cells during adipogenic differentiation. Images of Pmp70 immunofluorescence-labeled peroxisomes (**a**); scale bar, 25 µm (upper panel); 10 µm (lower panel). Forced miR-29a expression reduced peroxisomal granule formation (**b**), Pex14 (**c**), and Pmp70 (**d**) expression, whereas miR-29a knockdown promoted peroxisome formation. Mean ± S.E. is calculated from 3 experiments. * *p* < 0.05 upon ANOVA test and Bonferroni post hoc test. Scramble, scrambled control; miR-29a, miR-29a mimic; miR-29a-AS, miR-29a antisense oligonucleotide.

Furthermore, peroxisome is indispensable in fatty acid metabolism and transport during adipocyte formation [31]. We investigated whether miR-29a affected the peroxisome biogenesis of mesenchymal stem cells incubated in adipogenic media. miR-29a mimetic transfected cells showed few peroxisomes in the cytoplasmic compartment as evident from weak peroxisomal membrane protein 70 (Pmp70) immunofluorescence (Figure 6a) together with significant decreases in peroxisomal granule formation (Figure 6b) and peroxisome markers, including peroxisomal biogenesis factor 14 (Pex14; Figure 6c) and Pmp70 expression (Figure 6d) as compared to scrambled control-transfected cells. Plenty of peroxisomes and increased Pex14 and Pmp70 expression were present in miR-29a antisense oligonucleotide-transfected cells.

### 2.7. miR-29a Signaling Downregulated Leptin Expression

**Figure 7:**
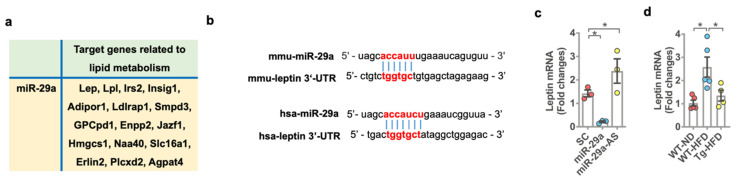
Effects of forced miR-29a expression or knockdown on leptin expression in mesenchymal stem cells and HFD-fed bone tissue. Bioinformatics prediction showing miR-29a targeted genes, which involved lipid or adipocyte metabolism (**a**). Sequences of 3′-UTR in human and murine leptin targeted by miR-29a (**b**). Gain of miR-29a function inhibited leptin expression, whereas miR-29a knockdown promoted the expression (**c**). miR-29a overexpression attenuated HFD-induced leptin expression in bone tissue (**d**). Mean ± S.E. of in vitro and in vivo models are calculated from 3 experiments and 4–5 mice, respectively. * *p* < 0.05 upon ANOVA test and Bonferroni post hoc test. Scramble, scrambled control; miR-29a, miR-29a mimics; miR-29a-AS, miR-29a antisense oligonucleotide; ND, standard rodent chow; HFD, high-fat diet.

Bioinformatics (david.ncifcrf.gov, accessed on 10 February 2021) predicted that miR-29a would target a plethora of genes, which involve adipocyte differentiation or lipid metabolic process (*p* < 0.01; Figure 7a). Of genes, leptin (lep) regulates adipose tissue metabolism and energy expenditure. miR-29a is predicted to target 3′-untranslated regions (3′-UTR) of human and murine leptin to interrupt the transcription (Figure 7b) (http://miRDB.org; http://www.targetscan.org, accessed on 10 February 2021). We revealed that forced miR-29a expression significantly inhibits leptin expression, whereas miR-29a knockdown promotes the expression (Figure 7b) in bone marrow mesenchymal cells. In bone tissue, leptin expression was significantly upregulated in HFD-fed WT mice. miR-29a overexpression compromised HFD-induced leptin expression (Figure 7c).

## 3. Discussion

Osteoporotic skeleton correlates with the development of obesity [32], excess glucocorticoid [33], and estrogen deficiency [34]-mediated marrow fat and visceral adipose accumulation. The molecular mechanism by which osteoporotic bone tissue dysregulates systemic adipose development warrants investigations. This study, for the first time, revealed the protective effects of miR-29a signaling on bone tissue integrity, which was advantageous in reversing HFD-induced energy underutilization and visceral fat overproduction. The microRNA repressed fatty acid/lipid metabolism transcriptome and peroxisome biogenesis to steer mesenchymal stem cells away from adipocytes, as well as targeted leptin signaling to mitigate HFD-induced body adiposity. Collective evidence conveyed a new insight into the microRNA mechanism, which balanced bone mass integrity and adipose development.

We utilized bone tissue-specific miR-29aTg mice to examine what role the molecule may play in HFD-mediated bone loss and adipose overdevelopment. HFD-fed miR-29a mice showed moderate bone mass loss and mild marrow adiposity. Analysis agreed with previous studies, revealing that miR-29a overexpression in osteoblasts prevents estrogen deficiency-induced osteoporosis in mice [26] and mediates IL-32γ protection against bone loss [35]. Furthermore, HFD-mediated hyperglycemia, hypercholesterolemia, and body overweight were attenuated in miR-29Tg mice, hinting that fat production or energy metabolism may be changed in these animals.

The HFD-induced whole-body fat deposit was compromised in miR-29aTg mice. This effect correlated with energy utilization improvement as low respiratory quotient and heat underproduction were reversed in these animals. We speculated that two reactions might compromise HFD-mediated energy underuse in miR-29aTg mice. Bone-forming cells consume tremendous energy or fatty acid to maintain bone tissue anabolism [36,37]. The analysis of improved bone mineral density and trabecular morphology in miR-29aTg mice explained the requirement of considerable energy expenditure for preserving osteoblast function and microstructure integrity, which prevented HFD-induced bone mass loss. Expanding studies have revealed that miR-29a signaling regulates lipid metabolism in the muscles of diabetic mice [38] and delays fatty liver development in HFD-fed mice [39], as well as inhibits adipocyte formation of human adipose mesenchymal stem cells [40]. We uncovered mild marrow adiposis together with moderate visceral adipose deposit in miR-29aTg mice. On the other hand, brown adipose tissue is important to maintain body thermogenesis and bone homeostasis in a cold condition [41]. The gain in subscapular brown fat mass consolidated the assumption that miR-29a overexpression favors energy metabolism and heat production in HFD-fed animals.

Transcriptomic landscapes revealed gene transcription inhibition, including Irs2, Ppp2r1a, Vldlr, CD36, Apoa5, and Ghr, etc., which involved fatty acid and lipoprotein metabolism pathways in miR-29aTg bone marrow mesenchymal progenitor cells. For example, Irs2 [42] and Ppp2r1α [43] signaling pathways regulate adipocyte formation of bone marrow mesenchymal stem cells. Vldlr knockdown represses lipid accumulation and inflammatory reaction in adipocytes [44]. CD36 is an important fatty acid receptor for adipocytic activity [45]. Apoa5 promotes lipid metabolism in the development of obesity [46]. In vitro model of this study confirmed the repressing effects of miR-29a signaling to adipocytic activity, including peroxisome loss and lipid underproduction, of mesenchymal stem cells incubated in an adipogenic condition. Furthermore, miR-29a signaling was advantageous to brown/beige adipocyte marker expression. Of genes, Ucp-1 knockout mice show decreased cortical bone mass and low bone formation rate [47]. Pgc-1α knockout accelerates adipocyte formation of mesenchymal progenitor cells [48]. This study shed light on miR-29a signaling inhibition of adipocytic activity in bone tissue. Profound investigations may extrapolate to the analysis of mild body fat mass in HFD-fed miR-29aTg mice. The changes in marrow adipocytic activity also indicated the complex nature of HFD-induced bone and fat imbalance.

Bioinformatics (http://miRDB.org; http://www.targetscan.org, accessed on 10 February 2021) predicts leptin as one of the miR-29a targets. The molecule is important to energy metabolism, obesity development, and diet-induced bone deterioration. High serum leptin levels correlate with the development of osteoporotic skeleton [49] and the risk of osteoporotic fracture [50] in menopausal women. In experimental animals, bone loss together with high serum leptin expression are present in HFD-fed mice [51] and methylprednisolone-treated animals [52]. We revealed that leptin was one of the molecular mechanisms underlying miR-29a control of systemic adiposity as HFD-induced leptin signaling was compromised in miR-29aTg bone. Serum or exosomal miR-29a involves the development of obesity or HFD-induced diabetes [53,54]. Other groups and we have revealed that low serum miR-29-3p or miR-29a levels are present in aged patients with osteoporosis [55] or postmenopausal women with osteoporotic spine fracture [56]. The limitations of this study, we should acknowledge, are that we do not exclude miR-29a signaling in osteoblasts that may directly or indirectly influence adipose tissue metabolism. The impact of bone tissue miR-29a on human obesity warrants investigations. Collective analysis of miR-29a-mediated transcriptomic landscapes for lipid metabolism also indicates multiple pathways may involve the interplay of the skeleton and systemic adipose homeostasis.

Taken together, miR-29a in osteoblasts maintained bone integrity, which reversed HFD-mediated energy underutilization and whole-body adipose overdevelopment. The microRNA regulated lipid metabolism and repressed leptin signaling to control adipocyte formation. This study highlights a new epigenetic mechanism underlying bone tissue protection against HFD-induced energy expenditure and fat overproduction.

## 4. Materials and Methods

### 4.1. Osteoblast-Specific miR-29a Transgenic Mice (miR-29Tg)

Animal experimentation protocols were approved by IACUC, Kaohsiung Chang Gung Memorial Hospital (IACUC Affidavit #2019091103). miR-29aTg mice with B57L/B6 background under control of osteocalcin promoter-driven miR-29a were utilized, and PCR genotyped, as previously described [26].

### 4.2. High Fed Diet Feeding

Three-month-old male miR-29aTg mice and age-matched male wild-type (WT) mice were provided with standard rodent chow (ND; 10 kcal%fat; D12450K, Research Diet Inc. New Brunswick, NJ, USA) or high-fat diet (HFD; 60 Kcal%fat; D1294, Research Diet Inc. New Brunswick, NJ, USA) [13] and water ad libitum for 6 consecutive months. Upon feeding, animals were euthanatized using an overdose anesthetic. Femurs, tibiae, and visceral and subscapular fat tissue were dissected, weighed, and normalized with body weight. Blood glucose, triglyceride, and cholesterol were examined using Biochemistry-SP-430 (Arkray Inc., Tokyo, Japan) for small animals, according to the manufacturer’s instructions.

### 4.3. In Vivo Metabolism Assay

An Automated Home Cage Phenotyping System (Phenomaster, TSE Systems, Homburg, Germany) [57] was utilized to characterize metabolic profiles of animals, according to the manufacturer’s manuals. In brief, each animal lived individually in a metabolic cage with free access to feed and water for 4 days. The cage was put in a 23 °C and 12 h dark/12 h light cycle climate chamber (TSE Systems, Homburg, Germany). Upon acclimation, 24 h (from 6:00 a.m. to 6:00 p.m.) O_2_ consumption (VO_2_), CO_2_ exhalation (VCO_2_), respiration quotient (RQ), feed, and water consumption by animals were automatically measured and normalized with body weight and feed consumption using High Throughput Physiological Signaling Calculation Unit and software of the system. Heat production (kcal/kg/h) is calculated automatically using the equation (3.941 × VO_2_ + 1.106 × VCO_2_)/1000, according to the maker’s instructions.

### 4.4. µCT Assessment of Whole-Body Adipose Deposit

The whole-body fat of each mouse was quantified using an 1176 Skyscan scanner (Bruker, Billerica, MA, USA) [27], according to the maker’s manual. In brief, animals were anesthetized using inhaled isoflurane, and 360° X-ray scanned (X-ray filter, 1 mm AI, X-ray intensity, 50 kV; pixel size 35 µm, rotation step, 0.9°; and frame averaging, 2). Images were reconstructed using SKYSCAN^®^ CT-Analysis software (smoothing kernel radius, 5; ring artifact reduction, 4; beam harden correction, 30). Images of adipose tissue of volume of interest (VOI) were binarized with a threshold range (73–96). Total volume, object volume, and structure thickness were calculated automatically.

### 4.5. µCT Analysis of Bone Microstructure

Microarchitecture and bone mass of skeletal specimens were scanned using μCT system (X-ray intensity, 50 KV, 500 μA, and 69 ms) to capture 400 slices of 9 μm × 9 μm × 9 μm radiographs, as previously described [26]. Radiograph reconstruction and bone mineral density (g/cm^3^) of specimens were quantified using SKYSCAN^®^ CT-Analysis software.

### 4.6. Histology

Morphology of the 5 μm tibia section was investigated using von Kossa staining protocol (NC9239431; Thermo Fischer Scientific, Waltham, MA, USA). Trabecular volume and tissue volume were measured using Zeiss microscope and image analyzer (Carl Zeiss AG, Jena, Germany), as previously described [26]. Morphology and number of marrow adipose were examined upon conventional hematoxylin and eosin staining protocols (NC0510871; Thermo Fischer Scientific, Waltham, MA, USA). Three regions of interest of each section and three sections of each animal were selected for histomorphometry.

### 4.7. Transcriptome Assay

Bone marrow mesenchymal cells were harvested from miR-29aTg mice and WT mice and incubated in DMEM and 10% fetal bovine serum, as previously described [58]. Upon Tri regent extraction of total RNA, aRNA in the specimen was amplified (Thermo Fischer Scientific Inc., Waltham MA, USA), labeled with CyDye (Cytiva, Malborough, MA, USA). Transcripts in specimens were probed using MOA2.0 Microarrays (Phalanx Biotech In, San Diego, CA, USA) and GeneChip™ System 3000D (Thermo Fischer Scientific Inc., Waltham MA, USA). Rosetta Resolver Biosoftware^®^ was used to cluster readouts. KEGG pathways were categorized using Kobas bioinformatics engine (http://kobas.cbi.pku.edu.cn, accessed on 10 February 2021).

### 4.8. miR-29a Mimetic and Antisense Oligonucleotide Transfection

Murine bone marrow mesenchymal cells (GIBCO^®^-C57BL/6 immortalized; S1502-100; Carlsbad, CA, USA) were incubated in DMEM with 10% fetal bovine serum, according to the maker’s instructions. A total of 5 × 10^5^ cells/well (12-well plates; Corning^®^ Costar^®^ TC-Treated Multiple Well Plates; CLS3512; Aldrich-Sigma, St Louis, MO, USA) were transfected with a scrambled control, miR-29a mimetic or antisense oligonucleotide using Oligofectamine™ Reagent (Thermo Fischer Scientific Inc., Waltham, MA, USA), according to the manufacturer’s manual.

### 4.9. Osteogenic and Adipogenic Differentiation Assay

Murine bone marrow mesenchymal cells (10^5^ cells/well, 24-well plate) were incubated in an osteogenic medium (A1007201; Thermo Fisher Scientific Inc., Waltham, MA, USA) for 18 days. Mineralized extracellular matrices were probed using von Kossa staining [26]. For adipocyte formation, 10^5^ cells were incubated in adipogenic medium (A1007001; Thermo Fisher Scientific Inc., Waltham, MA, USA) for 15 days [22]. Adipocytes were probed using Nile red staining kit. In some experiments, a total of 10^5^ cells/well (24-well plates) were incubated in a differentiation medium for brown adipocytes (BADTM, Cosmo Bio Co., Ltd., Tokyo, Japan) and white adipocytes (WATDM, Cosmo Bio Co., Ltd., Tokyo, Japan) for 15 days, respectively, according to the maker’s instructions.

### 4.10. RT-PCR

Upon reversely transcribing total RNA [23], Runx2 (Forward: 5′-CCAGCAGCACTCCATATCTC-3′; reverse, 5′-CAGCGTCAACACCATCATTC-3′) [26], osteocalcin (5′-CAAGCAGGGAGGCAATAAGG-3′; reverse, 5′-CGTCACAAGCAGGGTTAAGC-3′), Lpl (forward: 5′-GAGGAGCAGCCACATGGTAT-3′; reverse: 5′-GCTCGTCCTCTGTGG TCTTC-3′), aP2 (forward: 5′-AAGAAGTGGGAGTGGGCTTT-3′; reverse: 5′-GCTCTTC ACCTTCCTGCTTT-3′), UCP-1 (forward, 5′-CGATGTCCATGTACACCAAGGA-3; reverse, 5′-TTGTGGCTTCTTTTCTGCGA-3′), Pgc-1α (forward, 5′-AAACGATGACCCTCC TCACACCAA-3′; reverse, 5′-ACTGCGGTTGTGTATGGGACTTCT-3′), P2x5 (forward, 5′-GGGGTTCGTGTTGTCTCTGT-3′; reverse, 5′-CACTCTGCAGGGAAGTGTCA-3′), PAT2 (forward, 5′-GTGTTTCTGGCGACAATTT-3′; reverse, 5′-GGGAGAAGATGGT GAGGACA-3′), Tcf21 (forward, 5′-GAGAACGGGTCTCCACAGAA-3′; reverse, 5′-GTG GTCTTGAGCCTGGAGAA-3′), Hoxc9 (forward, 5′-ACCTCCTAGCGTCCAGGTTT-3′; reverse, 5′-GCCGTAAGGGTGATAGACCA-3′), leptin (forward: 5′-GCTGACATTCCC ACGTTTT-3′; reverse: 5′-CTGGGCCTGGTAGTTGTTGT-3′), and β-actin (forward: 5′-GACGGCCAGGTCATCACTAT-3′; reverse, 5′-CTTCTGCATCCTGTCAGCAA-3′) transcripts in the templates were investigated using primers together with 2 × TaqMan^®^ Universal PCR Master Mix with ABI 7900 Detection System (Applied Biosystems, Foster City, CA, USA). Relative changes in designated genes were calculated using 2^−ΔΔCt^, as previously described [26].

### 4.11. Confocal Fluorescence Microscopy

Peroxisomes in cell cultures incubated in adipogenic medium were probed using SelectFX Alexa Fluor 488 Peroxisome Labeling Kit (S34201, Thermo Fischer Scientific Inc., Waltham, MA, USA). In brief, 5 × 10^2^ cells/slide were fixed, permeabilized, and probed using peroxisomal membrane protein 70 (Pmp 70) antibody conjugated fluorescence Alexa Fluor 488. Immuno-labeled peroxisomes were visualized using a laser confocal microscope (Olympus; Tokyo, Japan). Immuno-labeled peroxisome vacuoles in each field of interest, three fields each slide, and three slides each experiment were counted.

### 4.12. Bioinformatics Search

The putative miR-29a targets, which regulated lipid metabolic process or adipocyte formation, were searched using DAVID Bioinformatics Research 6.8 engines (http://david.ncifcrf.gov, accessed on 10 February 2021). Gene sequences of 3′-UTR in human and murine leptin, which was targeted by miR-29a, were searched using miRDB (http://miRDB.org and Targetscan (http://www.targetscan.org), accessed on 10 February 2021) bioinformatics engines.

### 4.13. Statistical Analysis

The differences of investigations among miR-29aTg mice and WT mice with or without ND and HFD feeding were analyzed using analysis of variance test and Bonferroni post hoc test, where significant difference was set at *p* < 0.05.

## Data Availability

The data that support the findings of this study are available on request from the corresponding author.
